# Endodontic Sealers and Innovations to Enhance Their Properties: A Current Review

**DOI:** 10.3390/ma18184259

**Published:** 2025-09-11

**Authors:** Anna Błaszczyk-Pośpiech, Natalia Struzik, Maria Szymonowicz, Przemysław Sareło, Maria Wiśniewska-Wrona, Kamila Wiśniewska, Maciej Dobrzyński, Magdalena Wawrzyńska

**Affiliations:** 1 Pre-Clinical Research Center, Wrocław Medical University, Karola Marcinkowskiego 1, 50-368 Wrocław, Poland; anna.blaszczyk-pospiech@umw.edu.pl (A.B.-P.); natalia.struzik98@gmail.com (N.S.); maria.szymonowicz@umw.edu.pl (M.S.); magdalena.wawrzynska@umw.edu.pl (M.W.); 2 Department of Biomedical Engineering, Faculty of Fundamental Problems of Technology, Wrocław University of Science and Technology, Wybrzeże Stanisława Wyspiańskiego 27, 50-370 Wrocław, Poland; 3 Centre for the Circular Economy, Łukasiewicz Research Network—Łódź Institute of Technology, Marii Skłodowskiej-Curie 19/27, 90-570 Łódź, Poland; maria.wisniewska-wrona@lit.lukasiewicz.gov.pl; 4 Department of Dental Surgery, Faculty of Dentistry, Wroclaw Medical University, Krakowska 26, 50-425 Wroclaw, Poland; kamila.wisniewska@umw.edu.pl; 5 Department of Pediatric Dentistry and Preclinical Dentistry, Faculty of Dentistry, Wroclaw Medical University, Krakowska 26, 50-425 Wroclaw, Poland; maciej.dobrzynski@umw.edu.pl

**Keywords:** endodontic sealers, modification, biocompatibility, silver compounds, chlorhexidine, essential oils, biopolymers

## Abstract

Endodontic sealers are crucial for achieving an effective root canal obturation, preventing reinfection and promoting long-term treatment success. This review categorizes sealers by chemical composition, including traditional types such as zinc oxide-eugenol, glass ionomer, silicone, methacrylate and epoxy resins, calcium hydroxide, and the latest bioceramic formulations. Each type is evaluated for its physicochemical properties, biocompatibility, sealing ability, antimicrobial activity, and clinical limitations. A significant focus is placed on recent research into modifications of these materials with antimicrobial agents, aimed at improving antibacterial properties, bioactivity, and sealing performance. Among these, chitosan emerges as the most promising additive due to its broad antimicrobial spectrum, low cytotoxicity, and enhancement of sealing capacity. While bioceramic sealers represent a notable advancement due to their bioactivity and favorable interaction with moist environments, concerns regarding potential neurotoxicity and retreatability remain. The article presents recent advancements in the enhancement of endodontic sealers through the incorporation of organic and inorganic additives. It further delineates key research priorities, particularly the integration of bioactive materials, nanotechnology, and naturally derived compounds, with an emphasis on their potential applications in pediatric endodontics. Overall, while contemporary sealers offer a wide range of benefits, continued innovation is needed to optimize their biological safety, mechanical performance, and clinical usability.

## 1. Introduction

Root canal treatment is a procedure involving the removal of the pulp from the pulp chamber and root canals, followed by their mechanical and chemical preparation and obturation. Endodontic treatment aims to eliminate microorganisms, necrotic tissue, and accumulated hard tissue debris from the root canal system of the affected tooth, while preserving the tooth in the oral cavity for as long as possible. Indications for performing root canal treatment include irreversible pulpitis, pulp necrosis, and failure of previous root canal therapy. Pulp inflammation may result from various etiological factors, such as trauma, chronic irritation, and advanced carious lesions. Teeth after root canal treatment require functional restoration according to clinical indications [1,2]. Failure to perform endodontic therapy, or doing so improperly, can lead to both unpleasant and serious health consequences for the patient. These include severe toothache and, in the case of pulp necrosis, the formation of a dental abscess or osteitis. The most severe complication of pulpal disease is oral sepsis, a potentially life-threatening condition [3].

Infections originating from maxillary teeth can spread and lead to various serious medical complications, such as purulent sinusitis, meningitis, brain abscess, orbital cellulitis, and cavernous sinus thrombosis. In contrast, infections stemming from mandibular teeth may result in Ludwig’s angina, parapharyngeal abscess, mediastinitis, pericarditis, subcutaneous emphysema, and thrombophlebitis [3,4].

As an alternative to root canal treatment, tooth extraction may be considered. However, premature loss of a tooth can lead to occlusal imbalance, migration of neighboring teeth, and overloading of adjacent structures. These changes may cause pain, temporomandibular joint disorders, difficulties with mastication, and even psychological or emotional distress [5].

**Figure 1:**
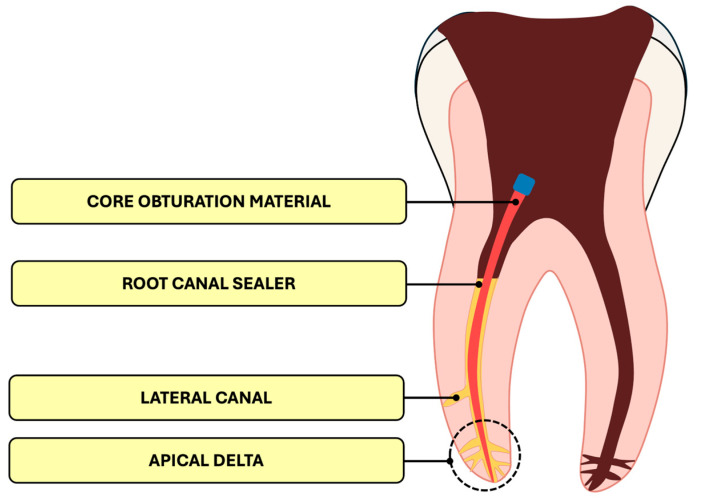
Internal structure of the root canal filling, including its components.

The process of root canal therapy consists of thorough chemical and mechanical preparation of the canal system, followed by obturation. Obturation involves sealing the apical region and filling the root canal space completely and void-free. Proper obturation prevents contamination and reinfection by saliva, bacteria, or periapical exudate, while also entombing residual microorganisms within the canal [6,7,8,9]. Root canal filling materials typically comprise a core material and a sealer [7]. The core material fills the bulk of the canal and serves as a barrier against reinfection—Figure 1. Although alternatives such as Resilon and the ProPoint system exist, gutta-percha remains the most widely used core material [7].

Gutta-percha is a naturally occurring thermoplastic substance derived from certain tropical trees native to South America and Malaysia [10]. It is manufactured into cones consisting mainly of gutta-percha and zinc oxide, along with plasticizers and radiopacifiers. Gutta-percha exists in two crystalline forms: alpha and beta. Upon cooling, it reverts to the beta phase, shrinking in the process—a drawback compounded by its lack of adhesion to dentin [11,12]. Nonetheless, gutta-percha offers advantages such as ease of handling, plasticity, radiopacity, and low toxicity [6,11].

Endodontic sealers are used to fill the spaces between the canal walls and the core material, as well as lateral and accessory canals—especially in the apical delta—where the core material alone cannot reach [13]. The sealer may also penetrate dentinal tubules, provided the smear layer has been adequately removed [14].

The viscosity of the sealer plays a crucial role in its effectiveness within the root canal system [15]. It is usually applied as a thin paste, facilitating the placement of core materials like gutta-percha cones [13]. In terms of obturation quality, the extent of sealer coverage along the canal walls may be more clinically significant than its maximum penetration depth [14].

Effective sealer placement depends on several factors, including the canal’s anatomy, cleanliness, and the degree of preparation [14,16]. A uniform layer of sealer should coat the canal walls before insertion of the core material [7,15]. Improper application may result in voids, microleakage, and treatment failure [17,18], while overextension of sealer beyond the apical foramen can impair periapical healing [19,20].

Various techniques for applying endodontic sealers have been described in the literature. These include the use of hand files (e.g., K-files), Lentulo spirals, paper points, gutta-percha cones, ultrasonic or sonic files, and pre-fabricated needle tips with syringes provided by manufacturers [7,14,15,16].

One widely used technique involves the Lentulo spiral, which is mounted on a low-speed handpiece, coated with sealer, and rotated to distribute the material within the canal. In a study by Kahn et al. [15], this method proved more effective than sonic files, K-files, and paper points. However, the technique carries a risk of instrument fracture and complications during retrieval if the spiral becomes lodged in the canal [6,16].

Seeking more reliable delivery methods, Tan et al. [16] developed a specialized sealer carrier consisting of Luer-lock needles of varying diameters. Their research demonstrated superior outcomes compared to the Lentulo spiral and hand spreaders. The larger-diameter needle acts like a “barrel,” allowing for more precise placement of the sealer and reducing accumulation in the coronal portion of the canal, which could otherwise hinder subsequent condensation of the paste [16].

The aim of this review is to provide an overview of commercially available endodontic sealers and recent research focused on enhancing their properties. This summary may help identify potential directions for future investigations in the field.

## 2. Classification of Endodontic Sealers

Grossman defined the characteristics of an ideal root canal sealer [21], which remain the benchmark for material evaluation to this day. According to his criteria, an optimal sealer should:

- Exhibit suitable viscosity upon mixing to ensure effective adhesion to the canal walls after setting;
- Form a hermetic seal;
- Be radiopaque to allow visualization on radiographic images;
- Be prepared as a very fine powder for ease of mixing with a liquid;
- Maintain dimensional stability and not shrink during setting;
- Avoid discoloration of the tooth structure;
- Be bacteriostatic or, at the very least, not support bacterial proliferation;
- Have a sufficiently slow setting time to allow for clinical manipulation;
- Be insoluble in tissue fluids;
- Be non-irritating to periapical tissues;
- Be soluble in a commonly available solvent to facilitate removal if necessary.

Despite ongoing advancements in material science, no single product currently fulfills all of these ideal criteria. Numerous endodontic sealers are commercially available, varying significantly in chemical composition and clinical performance.

In 2020, a strategic expansion of market activity in the United States prompted a comprehensive review by Komabayashi et al. [13], who analyzed the most widely used endodontic sealers in both the US and Japanese markets. Since the publication of that review, notable changes have occurred, including the discontinuation of several formulations and the introduction of new products driven by continued innovation and research. The quality of the sealant determines the long-term success of the treatment, including the prevention of recurrent infections and tooth preservation. Current technologies of endodontic sealer have significant limitations, like difficult conditions in the canals, lack of long-term stability, limited bioactivity, tissue compatibility, imperfect seal. Therefore, innovation remains essential because an ideal material that simultaneously exhibits optimal mechanical, biological, and chemical properties is still lacking and advancements in this field directly impact the effectiveness of endodontic treatment while reduction in complications [22,23].

This article discusses the following categories of endodontic sealers:

- Zinc oxide eugenol-based sealers;
- Zinc oxide-based sealers without eugenol;
- Glass ionomer-based sealers;
- Silicone-based sealers;
- Resin-based sealers (including methacrylate and epoxy resin formulations);
- Calcium hydroxide-based sealers;
- Bioceramic sealers.

Examples of root canal sealing products from the above-mentioned groups currently available on the dental market are presented in Table 1.

### 2.1. Zinc Oxide Eugenol-Based Sealers

Zinc oxide eugenol (ZOE)-based sealers are among the oldest and most extensively used materials in endodontics [13,44]. Standard formulations comprise two primary components: zinc oxide powder and eugenol liquid, an essential oil derived from clove plants. Upon mixing, a chelation reaction occurs, forming zinc eugenolate—a paste-like compound. In its set state, the material consists of zinc oxide particles embedded within a zinc eugenolate matrix. Free eugenol remains present during the setting process, gradually diminishing over time. The typical setting time for ZOE-based sealers ranges from 24 to 48 h [45,46,47].

These sealers are commercially available either as powder-liquid systems, such as Pulp Canal Sealer (Kerr, Brea, CA, USA) and Endomethazone N, or as two-paste systems like Tubliseal (Kerr, Brea, CA, USA). Two-paste formulations offer practical advantages, including longer working times and improved handling characteristics [12,48].

A notable feature of ZOE-based sealers is their strong antimicrobial activity, largely attributed to the presence of eugenol [49,50]. However, eugenol also exhibits cytotoxic properties, likely due to its effects on cellular respiration and membrane integrity. Despite this, cytotoxicity significantly decreases within 24 h after application, although low levels of eugenol continue to be released over time [45,51].

When extruded into periapical tissues, ZOE materials are resorbable through the action of blood flow, lymphatic drainage, and macrophages [52,53]. In cases requiring retreatment, removal of the sealer from the root canal system can be achieved using mechanical instrumentation in combination with solvents such as chloroform [54,55].

Despite their long-standing clinical use, ZOE-based sealers have several limitations. These include porosity and shrinkage, which contribute to dimensional instability and microleakage. The resulting gaps may permit the ingress of periapical fluids, increasing the risk of endodontic treatment failure [56]. Studies have documented measurable shrinkage and volume loss as early as three hours after mixing, attributed to the leaching of unreacted eugenol and hydrolysis of the zinc eugenolate matrix [57,58].

To address these drawbacks, eugenol-free formulations have been developed. As noted in the meta-analysis by Komabayashi et al. [13], this new class of sealers replaces eugenol with synthetic fatty acids as chelating agents, often combined with resins and zinc oxide. These fatty acid-based sealers are hydrophobic, a property that may enhance their biocompatibility and reduce microbial colonization within the root canal system [47,59]. Examples include Canals-N (Showa Yakuhin Kano, Tokyo, Japan) and Nogenol (GC, Alsip, IL, USA). However, due to their current unavailability, these products are not included in Table 1 of this review.

### 2.2. Glass Ionomer-Based Sealers

Glass ionomer-based sealers form a chemical bond with dentin through an ionic reaction between the calcium ions in dentin and the polyacrylic acid contained in the glass ionomer cement [60]. This interaction endows these materials with several advantageous properties, including minimal polymerization shrinkage—which reduces the risk of bacterial microleakage—low solubility in tissue fluids, and enhanced resistance to vertical root fracture [61,62,63,64].

The antibacterial activity of glass ionomer-based sealers is multifactorial. While their initial low pH contributes to this effect [65], the primary mechanism is the sustained release of fluoride ions, which inhibit bacterial growth. In a study by Shalhav et al. [66], the Ketac-Endo sealer exhibited strong, albeit short-lived, antibacterial properties.

However, the same features that contribute to the material’s durability—specifically, its chemical adhesion to dentin and high hardness—pose significant challenges during endodontic retreatment, particularly in efforts to remove the sealer from the root canal system [65].

### 2.3. Silicone-Based Sealers

Silicone-based sealers are composed primarily of divinyl polysiloxane and polymethylhydrosiloxane, which undergo curing via an addition reaction between vinyl and hydroxyl groups. Representative products from this group include GuttaFlow, GuttaFlow 2, RoekoSeal, and the most recent formulation, GuttaFlow Bioseal.

These materials are characterized by a short setting time—ranging from a few minutes depending on the formulation [9]—and low viscosity, which facilitates effective penetration into the root canal system [12]. They exhibit excellent biocompatibility and are non-cytotoxic, as demonstrated by Rodríguez-Lozano et al. [67], who found that both GuttaFlow 2 and GuttaFlow Bioseal maintained cell viability without inducing apoptosis.

Silicone-based sealers also offer reliable sealing performance due to their dimensional stability and virtually zero solubility in fluids [68,69,70]. Their formulation with silver particles prevents discoloration and corrosion while exerting a mild preservative effect within the root canal system [71]. However, a notable drawback is the lack of inherent antibacterial properties. Kapralos et al. [72] reported that GuttaFlow 2 and RoekoSeal were ineffective against biofilm formation by *Streptococcus mutans*, *Staphylococcus aureus*, *Staphylococcus epidermidis*, and *Enterococcus faecalis*.

The latest generation of these materials, GuttaFlow Bioseal, incorporates bioceramic components to enhance sealing ability and improve biocompatibility compared to earlier formulations. Notably, it promotes the differentiation of human periodontal ligament stem cells into cementoblast-like cells, even in the absence of growth factors [67].

Ruiz-Linares et al. [73] further demonstrated that GuttaFlow Bioseal exhibits superior antibiofilm and antibacterial activity at 1 and 4 weeks post-application when compared to the epoxy resin-based sealer AH Plus, whose antibacterial effect diminished over time. Additionally, GuttaFlow Bioseal has shown clinical potential for use in retrograde root fillings during apical surgery. The apical seal achieved with this material was found to be comparable to that of mineral trioxide aggregate (MTA), the current gold standard in such procedures [74].

### 2.4. Methacrylate Resin-Based Sealers

Methacrylate resin-based sealers can be classified into four generations, each representing advances in formulation and clinical performance.

The first generation, exemplified by Hydron, consisted of a gel based on 2-hydroxyethyl methacrylate and was unique in that it did not require a separate core filling material [75,76]. However, its use was discontinued in the 1980s due to several significant drawbacks, including difficulty of removal from the root canal system, induction of periapical inflammation, material resorption, high polymerization shrinkage, and water sorption [77,78].

The second generation includes sealers such as EndoRez (Ultradent Products, South Jordan, UT, USA), a hydrophilic urethane methacrylate-based material. It demonstrates good penetration into dentinal tubules, particularly following smear layer removal [79,80].

The third generation introduced systems incorporating self-etching primers and dual-cure composite resin sealers [81,82]. An example is FibreFill R.C.S., used with the FibreFill Primer System A and B. These systems achieve adhesion via micromechanical interlocking between the resin and dentin collagen, forming a hybrid layer that provides improved sealing and adhesive strength [83,84,85].

Another notable product in this category is Epiphany Root Canal Sealant (Pentron Clinical Technologies, Orange, CA, USA), which is designed for use with the Resilon core material instead of gutta-percha, enhancing bonding to root dentin. Its formulation includes ethoxylated glycidyl methacrylate, bisphenol A diglycidyl ether methacrylate (Bis-GMA), urethane dimethacrylate (UDMA), and hydrophilic bifunctional methacrylates [86]. Epiphany is a dual-cure sealer applied after dentin conditioning with 17% ethylenediaminetetraacetic acid (EDTA), and it is capable of forming covalent bonds with Resilon [87,88].

The fourth generation sought to simplify the application process by incorporating acidic monomers—previously used in separate primers—directly into the resin sealer. This innovation eliminated the need for separate etching and bonding steps, enabling a one-step approach that reduces application time and minimizes procedural errors [89]. Examples of this generation include MetaSEAL, containing 4-methacryloyloxyethyl trimellitate anhydride (4-META), and Super-Bond RC Sealer (Accel) (Sun Medical, Japan). Christos Gogos et al. [85] demonstrated that methacrylate resin-based sealers generally provide superior sealing and adhesion to root dentin compared to glass ionomer, epoxy resin, and calcium hydroxide-based sealers.

When combined with Resilon, methacrylate-based sealers also increase resistance to vertical root fracture, outperforming zinc oxide- and silicone-based sealers in this regard [90].

Despite their many advantages, methacrylate resin-based sealers are not without limitations. The most significant concern is their cytotoxicity. Studies have reported varying levels of toxicity—from moderate to severe—that tend to increase over time [91,92]. Additional drawbacks stem from the nature of the polymerization reaction itself. In the context of the root canal’s unfavorable geometry, polymerization shrinkage can lead to gap formation at the dentin-sealer interface. Moreover, residual monomers—left unreacted during curing—have been shown to be cytotoxic, inducing apoptosis in pulp and gingival cells, and may also possess genotoxic and mutagenic properties [77,93,94].

### 2.5. Epoxy Resin-Based Sealers

Epoxy resin-based endodontic sealers have long been regarded as the gold standard in root canal obturation due to their favorable physicochemical characteristics. These include low polymerization shrinkage, excellent sealing ability, dimensional stability, and low solubility—all of which contribute to minimizing reinfection and supporting long-term clinical success [95,96,97,98]. In addition, their extended working time and strong adhesion to root canal walls make them well-suited for both lateral compaction and thermoplastic obturation techniques [99].

The formulation of epoxy-based sealers typically includes an epoxy resin base combined with amines (as curing agents), zirconium dioxide (for radiopacity), as well as fillers such as calcite, silica, and various pigments [100,101]. The setting reaction involves a polymerization process between the epoxy groups and the amines, resulting in a highly cross-linked structure [102]. This reaction yields a material with high mechanical strength, exceptional sealing capacity, and minimal shrinkage—all factors that reduce the risk of microleakage [103].

One of the key advantages of these sealers is their ability to penetrate dentinal tubules, forming so-called resin tags that enhance mechanical retention and sealing at the dentin-sealer interface [104]. Moreover, the relatively slow polymerization rate allows for a prolonged working time, reducing the risk of premature setting during complex procedures [100].

Once cured, epoxy resin-based sealers form a dense, impermeable matrix that exhibits high resistance to chemical and mechanical degradation, even under moist conditions [105,106]. However, their low resorbability and lack of bioactivity may lead to complications if the material extends beyond the apical foramen. In such cases, the presence of sealer in periapical tissues can provoke a persistent inflammatory response due to the material’s limited ability to integrate or be resorbed biologically [77,107].

In terms of biocompatibility, epoxy-based sealers are generally classified as moderately biocompatible. While they may elicit a transient inflammatory response when in direct contact with periapical tissues, this reaction typically subsides over time [108]. However, in contrast to newer bioceramic materials—known for their bioactivity and ability to stimulate mineralization and tissue regeneration—epoxy resin sealers lack the capacity to induce the formation of structures such as hydroxyapatite [109].

Despite this limitation, their well-documented clinical performance and predictable physical properties continue to make epoxy resin sealers a reliable choice in contemporary endodontic practice [95].

A number of epoxy resin-based sealers are currently available on the market, each with slight variations in composition, handling, and clinical application. The most widely used is AH Plus (Dentsply Sirona, Charlotte, NC, USA), recognized for its excellent sealing properties, high radiopacity, good biocompatibility, and optimal working and setting times [97]. An alternative is Adseal (Meta Biomed, Chungcheongbuk-do, Republic of Korea), which offers similar physicochemical characteristics but with a slightly shorter setting time—an advantage in specific obturation techniques [110]. Another example, Topseal (Dentsply, Charlotte, NC, USA), shares a similar composition but exhibits higher viscosity compared to AH Plus, which may reduce its ability to penetrate narrow or highly curved canals [111].

### 2.6. Calcium Hydroxide-Based Sealers

Calcium hydroxide-based endodontic sealers constitute an important category of root canal obturation materials due to their favorable biological and physicochemical properties. Calcium hydroxide (Ca(OH)_2_) exhibits strong alkalinity (pH~12.5), which underlies its antimicrobial activity and ability to neutralize bacterial endotoxins. In addition, it promotes periapical healing by stimulating hard tissue formation [112]. These sealers are well known for their good biocompatibility and their osteogenic and cementogenic potential, making them particularly valuable in cases involving extensive periapical lesions or during endodontic retreatment procedures [113].

Several calcium hydroxide-based sealers are commercially available, with the most commonly used including Sealapex (Kerr, Brea, CA, USA), Apexit Plus (Ivoclar Vivadent, Schaan, Liechtenstein), CRCS (Coltene Whaledent, Altstätten, Switzerland), and Calapex (Prevest, Jammu, India). Sealapex, which incorporates calcium hydroxide into a resin-based matrix, is widely recognized for its high biocompatibility; however, it is also associated with a relatively long setting time and increased solubility over time [114]. Apexit Plus, combining calcium hydroxide with epoxy resin components, demonstrates good penetration into dentinal tubules and moderate solubility [115].

Although both in vitro and in vivo studies have shown that calcium hydroxide-based sealers generally exhibit lower long-term sealing ability compared to newer bioceramic materials, they are still valued for their bioactivity and their capacity to support tissue regeneration [116].

Despite certain limitations—such as greater solubility and potential for shrinkage over time—calcium hydroxide-containing sealers remain a justified and clinically relevant option, especially when the treatment objective extends beyond mere obturation to include biological stimulation of periapical healing. Typical indications include the management of periapical lesions, retreatment cases, and pediatric endodontic procedures, where material biocompatibility is a critical consideration [112,116,117].

### 2.7. Bioceramic Sealers

Bioceramic sealers represent the latest generation of root canal obturation materials, distinguished by their high bioactivity, biocompatibility, and favorable physicochemical properties. Their primary components are calcium silicates (CaSi), which, upon contact with moisture, undergo a hydration reaction resulting in the release of calcium and hydroxyl ions and the formation of hydroxyapatite at the interface with surrounding tissues [118,119,120]. This mechanism not only ensures a tight seal of the root canal system but also actively stimulates periapical healing and tissue regeneration [121]. Bioceramic sealers do not shrink during setting, are hydrophilic, and perform well in moist environments—qualities that set them apart from traditional epoxy- and calcium hydroxide-based sealers [122,123].

Mineral Trioxide Aggregate (MTA), one of the earliest calcium silicate-based materials, laid the foundation for the development of modern bioceramic sealers. MTA-based sealers demonstrate favorable bioactive and osteoinductive properties due to their ability to induce the formation of calcium apatite both in vitro and in vivo [119,124,125]. Their composition typically includes tricalcium silicate, dicalcium silicate, calcium oxide, and small amounts of aluminum and iron oxides, with bioactivity largely attributed to calcium ion release during silicate phase hydration [119,124,126].

Compared to traditional resin-based sealers, MTA materials exhibit lower cytotoxicity and better compatibility with periapical tissues [127]. They also offer mild antibacterial properties, linked to calcium ion release and an elevated pH environment [126]. In vitro studies have shown that MTA promotes fibroblast and osteoblast adhesion, facilitating periodontal tissue regeneration [124,125]. Despite these advantages, MTA’s extended setting time and challenging handling characteristics [124,126] have prompted the development of new bioceramic materials with improved clinical usability.

Modern bioceramic sealers retain the bioactivity and osteoinductive capabilities of MTA while offering better handling and shorter setting times. As such, they are increasingly regarded as the preferred choice for root canal sealing [127].

Several well-established bioceramic sealers are currently available on the market, including: EndoSequence BC Sealer (Brasseler), TotalFill BC Sealer (FKG, La Chaux-de-Fonds, Switzerland), BioRoot RCS (Septodont, Saint-Maurdes-Fossés, France), and AH Plus Bioceramic Sealer (Dentsply Sirona, Charlotte, NC, USA). EndoSequence BC Sealer is a premixed material requiring no mixing, featuring high radiopacity, a long working time, and excellent adaptation to canal walls due to its fine particle size (<2 µm) [109]. In contrast, BioRoot RCS is a two-component product based on tricalcium silicate, offering strong antibacterial and bioinductive properties. Studies have shown that BioRoot RCS induces cement formation and positively affects tissue mineralization [128]. Recent innovations also include bioceramic modifications of established sealers, such as AH Plus Bioceramic, which combines the chemical stability of resin-based materials with the biological activity of bioceramics. Bioceramic sealers such as EndoSequence BC Sealer (Brasseler, Savannah, GA, USA), BioRoot RCS (Septodont, Saint-Maurdes-Fossés, France), and TotalFill BC Sealer (FKG, La Chaux-de-Fonds, Switzerland) offer a unique combination of high bioactivity and excellent chemical and mechanical stability. They contain calcium silicate compounds that hydrate in the presence of moisture, forming a hydrophilic structure capable of releasing calcium and hydroxyl ions, promoting apatite formation on the material surface [129].

However, despite their high biocompatibility and favorable biological profile, bioceramic sealers are not entirely free from adverse effects. When inadvertently extruded beyond the apical foramen—particularly in anatomically sensitive areas such as the mandibular canal—they may provoke neurological complications. Entry of the sealer into the inferior alveolar nerve canal can result in paresthesia, neuropathic pain, or, in severe cases, permanent nerve damage [130,131,132]. These adverse effects are believed to stem from the high pH and the release of calcium and hydroxyl ions, which may irritate neural cells, including Schwann cells [133]. Additionally, some bioceramic materials exhibit slight volumetric expansion during setting, which—within the confined space of the mandibular canal—could exert mechanical pressure on the nerve [134].

Although in vitro studies have shown that bioceramic sealers such as EndoSequence BC Sealer and BioRoot RCS are less cytotoxic to neural cells than traditional epoxy-based materials, prolonged exposure at high concentrations can negatively affect the viability and morphology of glial cells [135]. For this reason, accurate working length determination and the use of minimal amounts of sealer are especially critical when treating mandibular molars and second premolars, whose root apices may be located in close proximity to the mandibular canal [131]. Awareness of the potential neurotoxicity of bioceramics is particularly important in cases involving periapical pathology (e.g., resorption or bone fistulas), where the bony barrier separating the root apex from the neurovascular bundle may be compromised.

## 3. Study Results on Modified Sealers

Advancements in dental material science have led to ongoing efforts to enhance the properties of conventional root canal sealers through the incorporation of various bioactive and antimicrobial particles. A key focus of this research has been the improvement of antibacterial efficacy, particularly in the context of persistent infections, which remain a major cause of endodontic treatment failure. Among the most clinically relevant pathogens associated with such failures are *Enterococcus faecalis* and *Candida albicans* [136,137].

*E. faecalis* is a facultative anaerobic Gram-positive bacterium with a remarkable capacity to survive under extreme conditions. It can colonize nutrient-deprived root canals, tolerate low pH environments, and resist the action of disinfectants [138,139]. Moreover, it is capable of forming robust biofilms, penetrating dentinal tubules, and evading the host immune response, which makes it particularly resistant to conventional endodontic cleaning and disinfection protocols [139]. As a result, *E. faecalis* is frequently isolated in cases of secondary endodontic infections and in teeth exhibiting chronic periapical inflammation [140].

*C. albicans*, on the other hand, is a dimorphic fungal organism capable of switching between filamentous and yeast-like forms. It is strongly associated with failed endodontic treatments and is implicated in the development of chronic periapical lesions [137]. This species demonstrates a high affinity for hydroxyapatite and readily adheres to dentin, enamel, and cementum—regardless of whether these tissues have been conditioned with EDTA. Additionally, *C. albicans* can bind to both type I and type IV collagen [141], enhancing its ability to persist within the root canal system. Its resilience is especially problematic in anatomically complex canal morphologies, where complete mechanical removal is difficult [142].

**Figure 2:**
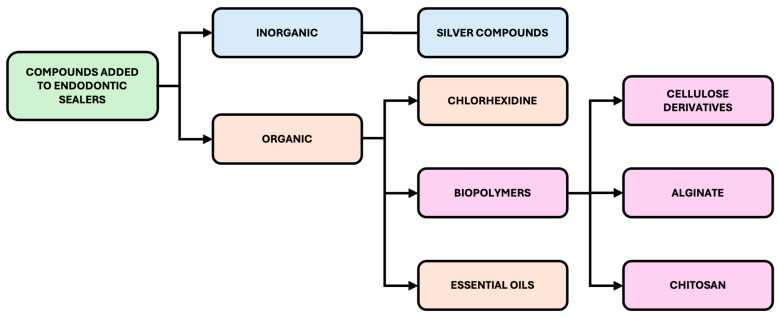
Classification of chemical compounds added to commercially available modified sealer formulations.

To address these challenges, recent studies have explored the incorporation of various antimicrobial agents into existing commercial sealer formulations. These modifications aim to improve the overall disinfection efficacy of the root canal obturation process. A classification of the chemical compounds currently investigated or applied for this purpose is presented in Figure 2.

### 3.1. Modification of the Sealers with Silver Compounds

Nanomodification of root canal sealers using nanostructured silver vanadate (AgVO_3_) decorated with silver nanoparticles has been extensively studied due to the well-documented antimicrobial properties of silver nanoparticles. Teixeira et al. [143] demonstrated that while all freshly prepared sealers—both modified and unmodified—effectively inhibited *E. faecalis*, significant differences in antimicrobial activity became apparent only after the materials had set. Specifically, Sealer 26 and Endomethasone N with 5–10% nanostructured AgVO_3_ exhibited significantly greater antimicrobial activity than their unmodified counterparts. AgVO_3_ exerts antimicrobial activity through a synergistic action of silver nanoparticles and vanadium nanowires, which induce oxidative stress and compromise bacterial membranes, leading to cell death [144,145].

The addition of nanostructured AgVO_3_ also influenced setting times—shortening them in AH Plus and prolonging them in calcium oxide- and eugenol-based sealers [144]. Follow-up studies by the same research group [144,145] revealed that AgVO_3_-modified sealers, particularly Sealer 26 and Endomethasone N, caused a notable reduction in human gingival fibroblast viability. In the case of AH Plus, cytotoxicity was moderate at 24 h but progressed to near-total cell death after 7 days [145].

Ionic analysis identified the release of Ag^+^ and V^4+^/V^5+^ ions as the primary mechanism of cytotoxicity [144,146]. Additionally, nanostructured AgVO_3_ negatively affected the esthetic properties of some sealers, causing long-term discoloration [147]. While the use of nanostructured silver vanadate can enhance the long-term antimicrobial effect of sealers, its clinical applicability remains limited due to cytotoxicity concerns and the need for further optimization of its concentration and formulation.

### 3.2. Modification of the Sealers with Chlorhexidine

Chlorhexidine (CHX) is among the most extensively studied antimicrobial agents in endodontics. Its incorporation into root canal sealers—in both molecular and encapsulated forms—has been shown to significantly improve activity against *E. faecalis*. At a physiological pH range of approximately 5.5 to 7.0—corresponding to the pH of human tissues—CHX is most effective because it dissociates from its salt form into a positively charged cation that binds to negatively charged microbial cell walls, disrupting osmotic equilibrium by increasing membrane permeability and causing leakage of low molecular weight cytoplasmic components at lower concentrations, while at higher concentrations, it induces precipitation and coagulation of cytoplasmic contents leading to microbial cell death [148].

In a study by Collares et al. [144], the addition of 2.5% and 5% CHX to resin-based sealers enhanced their antimicrobial performance. When combined with α-tricalcium phosphate (α-TCP), the formulation also promoted remineralization, potentially supporting periapical healing. However, higher CHX concentrations were found to compromise pH stability and increase material degradation, which may limit clinical applicability [149].

Carvalho et al. [150] explored the use of CHX nanoparticles complexed with hexametaphosphate (CHX-HMP NPs), which were incorporated into commercial sealers. This modification led to prolonged antimicrobial activity without negatively impacting key physicochemical properties. Among tested materials, MTA Fillapex containing 5% CHX-HMP NPs exhibited the highest cytotoxicity, while AH Plus showed the greatest biocompatibility.

In another study, Raddi [151] introduced liposomal CHX into the bioceramic sealer BioRoot RCS. Liposomal encapsulation significantly improved control over CHX release—extending antimicrobial activity up to sevenfold—while also reducing cytotoxicity compared to conventional CHX solutions. These findings suggest that carrier-based delivery systems, such as nanoparticles and liposomes, may offer a promising strategy to balance antimicrobial efficacy and biological safety.

### 3.3. Modification of the Sealers with Essential Oils

An alternative direction in the enhancement of root canal sealers involves the incorporation of essential oils, particularly those with known antimicrobial and anti-inflammatory properties. Although research in this area remains limited, promising results have been reported with essential oils derived from *Butia capitata* fruit and oleoresin obtained from the *Copaifera* tree (commonly known as copaiba oil resin). Reiznautt et al. provided evidence that experimental sealers with natural oil exhibit antimicrobial activity against *Enterococcus faecalis* [152].

*Butia capitata* oil has demonstrated antimicrobial activity attributed to the presence of medium- and long-chain fatty acids [153]. Copaiba, an oleoresin exuded from the trunks of *Copaifera* trees, has been extensively studied for its anti-inflammatory, analgesic, reparative, and antimicrobial effects. Copaiba oil exhibited bactericidal activity against *Fusobacterium nucleatum*, *Streptococcus mitis*, *Prevotella nigrescens*, *Porphyromonas gingivalis*, *Lactobacillus casei*, *Streptococcus salivarius* and *Streptococcus mutans* [154,155,156].

Based on these properties, Garrido et al. [157] developed an experimental endodontic sealer (Biosealer) using copaiba oil resin as the liquid component. The powder phase consisted of zinc oxide, calcium hydroxide, bismuth subcarbonate, and sodium tetraborate. Biosealer demonstrated favorable physicochemical characteristics—including setting time, flow, film thickness, dimensional stability, radiopacity, and solubility—meeting the requirements of ANSI/ADA Specification No. 57.

The research was further expanded to compare Biosealer with commercial products. Using osteoblast-like Osteo-1 cells, biocompatibility tests revealed that the copaiba-based sealer was non-cytotoxic. The authors attributed this to a potential acid-base reaction between the acidic copaiba components and the alkaline powder constituents, resulting in the formation of mildly irritating but biocompatible salts [157].

In addition to copaiba, Reiznautt et al. [152] investigated the use of *Butia capitata* oil in experimental formulations. Groups containing natural oils exhibited higher degrees of monomer conversion and lower water sorption and solubility compared to commercial methacrylate-based resin sealers. Furthermore, these formulations resulted in reduced fibroblast cell death, supporting their potential as biologically safer alternatives.

### 3.4. Modification of the Sealers with Biopolymers

Polymers have long played a key role in dentistry, with applications in filling materials, cements, prosthetic components, and adhesive systems [158]. A polymer is a chemical compound composed of repeating structural units—monomers—linked by chemical bonds into long molecular chains. The process of joining monomers to form polymers is known as polymerization [159,160]. Depending on their molecular architecture, polymers can be linear, branched, or cross-linked, which influences their physical and mechanical properties. Based on their origin, polymers are classified into synthetic (e.g., polymethyl methacrylate—PMMA, widely used in denture bases) and natural biopolymers, such as alginates, cellulose, and chitosan [160].

The ability to tailor their physicochemical characteristics makes polymers particularly attractive in dentistry, enabling the development of materials that combine mechanical strength, esthetics, and biocompatibility [160]. Their elasticity, strength, biodegradability, and compatibility with biological tissues have led to their widespread use in dental and biomedical applications. Recent advances in polymer science have also enabled the creation of smart materials that respond dynamically to environmental changes in the oral cavity [161].

Natural biopolymers—biodegradable materials of biological origin—are gaining increasing importance in dentistry due to their inherent biocompatibility and regenerative potential [162,163]. They are currently employed in drug delivery systems, bone regeneration membranes, and wound healing materials [164]. Their ability to minimize inflammatory responses and support repair processes further underlines their clinical relevance [164]. Ongoing research aims to enhance their mechanical durability and optimize the controlled release of bioactive agents to accelerate tissue regeneration [164].

#### 3.4.1. Alginate

Alginate is a naturally occurring, anionic, hydrophilic polymer primarily extracted from brown seaweed (*Phaeophyceae*) and produced by certain bacteria, including *Azotobacter vinelandii*, *Pseudomonas aeruginosa*, and *Pseudomonas fluorescens* [165]. It is valued for its favorable properties: biocompatibility, biodegradability, low toxicity, resorbability, ease of handling, and cost-effectiveness. Importantly, its surface chemistry supports cell adhesion and proliferation [166].

Alginate’s ability to form hydrogels allows for controlled drug delivery at target sites [167], while its semi-permeable structure promotes cell attachment, proliferation, and differentiation—making it a promising scaffold for tissue engineering [168]. Although alginate is widely used in dental materials, its application in endodontic sealers is still limited in the literature. However, a study by Huang et al. [169] demonstrated that a bioactive glass-based sealer modified with 1% sodium alginate and zirconium oxide exhibited excellent flowability, appropriate film thickness, radiopacity, and rapid setting time. The modified material also showed strong biocompatibility, good sealing ability, and mineralization potential—supporting its potential for clinical endodontic use [169,170].

#### 3.4.2. Cellulose Derivatives

Cellulose and its derivatives—particularly methylcellulose and hydroxypropyl cellulose—have been evaluated as components of endodontic materials. These materials inherently lack antimicrobial properties [171]. Cellulose is a plant-derived polysaccharide synthesized through photosynthesis and represents the most abundant natural polymer on Earth. It serves a structural role in plants and can be obtained from sources such as wood, cotton, flax, and hemp, which differ in purity and cellulose content [172].

Hydroxypropyl cellulose is a semi-crystalline polymer with low glass transition temperature, offering high molecular mobility and plasticity [173]. Methylcellulose is derived from cellulose through alkaline treatment followed by methylation. It is a non-toxic compound with stabilizing, film-forming, and thickening properties, commonly used in food and cosmetic products [174,175].

Baba et al. [176] investigated the effects of incorporating these cellulose derivatives into pre-mixed calcium silicate-based cements, such as MTA. The addition of low-viscosity methylcellulose and hydroxypropyl cellulose improved flow characteristics and extended setting times, with methylcellulose having a more pronounced effect on the latter. However, both modifications resulted in reduced calcium ion release from the cement [176]. As calcium ions are essential for hard tissue formation via ATP-dependent reactions [177] and for immunomodulatory functions [178], this reduction may affect the material’s bioactivity and clinical performance.

#### 3.4.3. Chitosan

Chitosan (CS), poly(b-(1,4)-2-amino-2-deoxy-D-glucopyranose), is the primary derivative of chitin—the second most abundant natural polysaccharide after cellulose. Chitin is primarily found in the exoskeletons of marine crustaceans (e.g., crabs, shrimp), insects, and the cell walls of fungi, yeasts, and molds [179]. Chitosan is characterized by a broad spectrum of antibacterial activity, effective against both Gram-positive and Gram-negative bacterial strains [180]. According to literature data supported by research, the antibacterial and antifungal activity of chitosan is attributed, among other factors, to its positively charged amino groups, which interact with the negatively charged lipopolysaccharide and protein groups on the surface of microbial cells, leading to membrane disruption and damage to the bacterial cell wall [181].

CS is extensively used across various dental fields. In preventive dentistry, it appears in toothpastes and mouthwashes. In conservative dentistry, it serves as a pulp capping agent. In implantology, it is used as a component of titanium coatings. It also plays a role in surgical dressings, bone graft substitutes, drug delivery systems, and guided tissue regeneration. It is also part of photodynamic therapy against *Porphyromonas gingivalis* (*P. gingivlis*) in periodontology. Furthermore, CS enhances the antimicrobial properties of restorative and adhesive materials. In endodontics, it facilitates sustained calcium ion release from calcium hydroxide formulations, supports smear layer removal, and is a component of intracanal medicaments targeting *E. faecalis* and *C. albicans* [182,183,184].

CS is particularly noted for its antimicrobial activity. Ratih et al. [185] showed that incorporating CS into endodontic sealers enhanced antibacterial efficacy. The addition of CS nanoparticles to epoxy resin-based sealers significantly increased their activity against *E. faecalis*, while reducing cytotoxicity compared to unmodified formulations. Similarly, Maharti et al. [186] developed a novel tricalcium silicate–CS sealer, which showed negligible cytotoxicity to fibroblast cells and outperformed both epoxy and calcium silicate-based sealers in terms of biocompatibility.

Given the clinical relevance of *C. albicans* in endodontic failures, antifungal activity is a key property of sealers. Studies by Pattanaik et al. [187] demonstrated that sealers containing 2% CS exhibited significantly improved antifungal efficacy compared to standard sealers. Notably, CS-enhanced epoxy resin sealers were more effective against fungal pathogens than those based on calcium hydroxide or MTA.

CS also improves sealing ability by enhancing adhesion to dentinal tubules. Rane et al. [188] reported that adding nanochitosan improved the sealing capacity of bioceramic sealers through better dentin diffusion and tubule penetration. Harishma et al. [189] further confirmed that 2% CS increased the push-out bond strength of calcium silicate-based sealers, surpassing even epoxy resin-based materials.

CS’s greatest potential lies in its use as a carrier for antimicrobial agents, such as CHX or silver nanoparticles (AgNPs). In an in vitro study [190], a sealer modified with CS nanoparticles (CS-NPs) and CHX showed the highest antibacterial activity, surpassing that of sealers modified with CHX, AgNPs, or calcium hydroxide alone. The most significant improvements were observed in Tubliseal and AH Plus, with antibacterial indices increasing by 40% and 32.7%, respectively. SEM analysis revealed significantly reduced colonization of *E. faecalis* on CS-NPs–CHX-modified surfaces. Additionally, CS enhanced the structural stability of nanoparticles without negatively affecting key physicochemical properties such as pH or solubility. Although cytotoxicity was not directly assessed, the high zeta potential (+52 mV) indicated excellent colloidal stability and potential for further clinical development—Table 2.

## 4. Discussion

Root canal sealers are a key component of effective endodontic treatment, ensuring the tight seal of the obturation and reducing the possibility of reinfection of the root canal system. A review of commercially available sealers reveals considerable diversity in terms of chemical composition, physicochemical properties, and biological behavior, allowing clinicians to select materials based on specific clinical conditions and individual operator preferences—Table 3.

Zinc oxide eugenol-based sealers exhibit strong antimicrobial properties [49] due to the presence of eugenol. However, studies indicate significant cytotoxicity associated with this compound [45]. Additionally, once set, the material becomes porous and undergoes considerable polymerization shrinkage, leading to the formation of microleakage [56]. The material is resorbable if extruded beyond the apical foramen.

Glass ionomer sealers, owing to their fluoride content, demonstrate strong antibacterial activity [66], relatively low post-setting shrinkage, and increased resistance to vertical root fracture. They also form a chemical bond with dentin [66], which ensures a good seal but complicates removal from the canal system in cases of endodontic retreatment [62].

Silicone-based sealers exhibit favorable physicochemical and mechanical properties. Their low viscosity [12] allows for excellent penetration into the root canal system. They are dimensionally stable, insoluble, and non-cytotoxic [68,69,70]. However, they have a major disadvantage in the form of a lack of antibacterial properties, which are absolutely necessary considering that root canal treatment is usually caused by a bacterial infection of the root system [72]. The exception at this point seems to be Guttaflow Bioseal, a bioceramic-modified silicone sealer that exhibits good antibacterial and anti-biofilm activity [73].

Methacrylate resin-based sealers offer superior adhesion to dentin compared to sealers based on glass ionomer, epoxy resin, or calcium hydroxide [85]. In combination with Resilon, they show increased resistance to vertical root fractures compared to zinc oxide-eugenol and silicone-based sealers [90]. However, in cases of complex root canal anatomy, gap formation and the presence of residual monomer have been reported [78]. The residual monomer is highly cytotoxic and has also been associated with genotoxic and mutagenic effects [93].

Epoxy-based endodontic sealers are among the most commonly used materials in dental practice. They exhibit favorable physicochemical properties, such as low polymerization shrinkage [103], good sealing ability, and dimensional stability [106]. These sealers also demonstrate high biocompatibility [108] and low solubility, which reduces the risk of reinfection. However, when extruded beyond the apical foramen, they may cause chronic inflammation due to limited bioactivity and slow resorption. These reactions usually subside over time [108,111].

Compared to modern bioceramic materials, which exhibit bioactive properties and can stimulate tissue regeneration, epoxy sealers do not induce the formation of new mineral structures such as hydroxyapatite [95]. Nevertheless, their predictable physical properties and clinically documented efficacy mean that they remain widely used in root canal treatment [107].

Calcium hydroxide-based sealers have strong tissue regeneration properties, especially in hard tissues such as bone, dentine and cementum. These sealers are especially recommended in cases involving large periapical lesions or re-endodontic treatments, where the therapeutic objective extends beyond simple canal obturation [113] to include the stimulation of periapical tissue repair, an aspect particularly important in pediatric patients. Although they demonstrate superior biocompatibility parameters, they may be less durable in terms of long-term sealing ability [191].

Compared to conventional epoxy-based sealers such as AH Plus (Dentsply Sirona, Charlotte, NC, USA), calcium hydroxide-based sealers demonstrate superior biocompatibility and biological activity. However, they are inferior in terms of long-term sealing ability and resistance to solubility. AH Plus, due to its stable, water-insoluble structure and excellent adhesion to dentin, remains the gold standard in many clinical practices, particularly in cases requiring durable and hermetic canal obturation [95].

In contrast to traditional calcium hydroxide-based sealers, bioceramic materials offer lower solubility and improved sealing properties, and are particularly well-suited to the moist environment of the root canal system [116].

The most recent group of endodontic sealers comprises bioceramic materials, which provide both a tight seal of the root canal system and stimulate the regeneration of periapical tissues [121]. Additionally, they exhibit low polymerization shrinkage and are well tolerated in moist environments [123]. However, studies have indicated their potential neurotoxic effects, so particular caution is advised when working in proximity to the inferior alveolar nerve [131].

Compared to epoxy-based sealers, bioceramics demonstrate lower cytotoxicity and reduced solubility. In comparison to calcium hydroxide-based sealers, they offer better volumetric stability and enhanced clinical durability [103]. Importantly, bioceramic sealers are capable of chemical bonding to dentin through the formation of so-called biomineralized tags, which may contribute to improved long-term sealing of the obturation [129].

However, their limitations include difficult removal during retreatment procedures and relatively higher cost of the materials.

The modification of currently available commercial endodontic materials through the integration of antimicrobial agents such as chlorhexidine, silver, and chitosan represents a promising direction in the development of root canal sealers with prolonged and targeted antibacterial activity. Each of the strategies investigated has its advantages and limitations: chlorhexidine, especially in encapsulated form, is distinguished by its balance between efficacy and safety; silver, although effective in inhibiting the growth of microorganisms, is characterized by significant cytotoxicity; whereas chitosan serves as a universal, biocompatible carrier that enhances the performance of active compounds.

The addition of nanostructured silver vanadate improved the antibacterial properties of sealers, although it affected the setting time differently depending on the chemical composition of the sealer [143,181]. The release of silver and vanadium ions was observed, resulting in reduced viability of human gingival fibroblasts [144,145]. The antimicrobial efficacy of sealers modified with chlorhexidine, particularly against *E. faecalis*, was found to be higher compared to their unmodified counterparts [149]. In the case of sealers supplemented with copaiba and butia oil resin, lower fibroblast mortality was observed in comparison with commercial products [152].

Moreover, sealers containing essential oils demonstrated a higher degree of conversion and lower water sorption [152]. The effect of sodium alginate on sealer properties remains inconclusive. It exhibited acceptable sealing ability, minimal cytotoxicity, and excellent biocompatibility. Its application in endodontic sealers may prove beneficial, but further research is needed to confirm its efficacy and safety [170,171]. Cellulose derivatives, when incorporated into sealers, resulted in increased flowability; however, they also had undesirable effects, including prolonged setting time and reduced calcium ion release from calcium silicate-based cements [176].

Chitosan emerged as the most promising biopolymer with the most favorable properties. In many studies, it improved antibacterial properties, especially against *E. faecalis* and *C. albicans*, which are common causes of endodontic treatment failure [185,187]. Chitosan also improved the physical properties of the sealers, including increased bond strength, thereby contributing to the reduction in apical microleakage. These effects have been confirmed in in vitro studies [188,189].

The incorporation of various antimicrobial agents into root canal sealers demonstrates significant potential in enhancing their efficacy against persistent endodontic pathogens, particularly *E. faecalis* and *C. albicans*. However, findings across studies indicate that antimicrobial effectiveness is highly dependent on both the type of modifying agent and its concentration. Silver-based modifications, especially with nanostructured AgVO_3_, showed sustained antibacterial activity even after sealer setting, suggesting a long-term benefit over unmodified formulations [143]. Yet, the associated cytotoxicity and esthetic drawbacks [144,145,146,147,148] raise concerns regarding their clinical applicability, highlighting the need for careful optimization of ion release and dosage. Conversely, chlorhexidine (CHX)-based modifications appear to balance antimicrobial performance with improved biocompatibility, particularly when delivered via nanoparticles or liposomes [149,150,151]. Such controlled-release systems offer prolonged antimicrobial activity while minimizing cytotoxic effects, representing a promising direction for future formulations. Essential oil-based sealers, including those incorporating copaiba and *Butia capitata* oils, show encouraging antimicrobial properties coupled with reduced fibroblast toxicity [152,157], although evidence remains limited and largely experimental. Among all modifications, chitosan (CS) emerges as a particularly versatile additive due to its intrinsic antibacterial and antifungal activity and its synergistic role as a carrier for agents like CHX and silver nanoparticles [186,187,188,189,190,191]. Sealers modified with CS consistently demonstrated enhanced antimicrobial efficacy while maintaining or improving biocompatibility and sealing ability, suggesting that biopolymer-based delivery systems may provide the most balanced strategy. Collectively, these findings underscore that while antimicrobial enhancement is achievable through diverse modifications, clinical translation requires a nuanced approach integrating efficacy, safety, and material stability.

Endodontic treatment of primary teeth, although significantly less common than in permanent teeth, is sometimes necessary, particularly in cases of irreversible pulp damage. In such scenarios, special resorbable endodontic materials are employed, including zinc oxide-eugenol-based pastes, iodoform-containing preparations, calcium hydroxide pastes (with or without iodoform), as well as mineral trioxide aggregate-type materials [192]. Although most clinical data confirm their effectiveness, some in vitro studies have shown a lack of antimicrobial activity, which may be related to chemical reactions between the components of the preparation [192]. A systematic review of the literature conducted by Barja-Fidalgo et al. [193] aimed to evaluate the effectiveness of alternative materials for filling root canals in deciduous teeth compared to ZOE cement. The authors demonstrated that both ZOE and pastes containing iodoform and/or calcium hydroxide, such as Vitapex, achieved high clinical and radiographic success rates, with the differences between them generally being small and statistically insignificant. ZOE may remain in the tissues due to slower resorption, while resorbable pastes may require retreatment. Due to the methodological limitations of the available studies, the authors emphasize the need for further high-quality randomized clinical trials with longer follow-up periods.

The root canal filling materials for primary and permanent teeth must meet different requirements, primarily due to differences in tooth morphology and the need for timely, controlled resorption of the filling. The development of an ideal material poses a challenge for researchers, as it must be capable of resorbing concurrently with the physiological root resorption of the primary tooth, biocompatible with periapical tissues and the developing permanent tooth germ, and resorbable in cases of extrusion beyond the apex [194].

## 5. Future Perspectives

Technological advancements and the ongoing development of biomaterials are opening new avenues for designing endodontic sealers with improved properties, including enhanced adhesion, reduced cytotoxicity, antimicrobial activity, and even the ability to stimulate tissue regeneration. Numerous studies are being conducted using commercially available sealers in combination with various organic and inorganic compounds to improve their overall performance.

While classical, well-established sealers—such as those based on zinc oxide-eugenol or epoxy resin—remain in use, there is a clear shift toward next-generation materials, particularly bioceramic sealers. These are gaining popularity due to their bioactivity, compatibility with the moist environment of the root canal, and their capacity to promote tissue regeneration. However, despite their advantages, several studies have raised concerns about the potential neurotoxicity of some bioceramic formulations [67,131]. Therefore, future research should not only aim to eliminate these undesirable properties but also focus on improving their mechanical characteristics, optimizing setting times, and enhancing their removability in cases requiring endodontic retreatment [122].

Investigating modifications in the composition of bioceramics is becoming increasingly important, as it may lead to the development of materials with more predictable biological behavior, improved compatibility with periapical tissues, and controlled release of bioactive substances [95]. A particularly promising direction for future studies involves the integration of bioceramics with nanotechnology and naturally derived compounds to enhance their functionality and clinical safety.

Another compelling avenue of research is the combination of conventional endodontic sealers with biopolymers, which offer unique properties such as biodegradability, biocompatibility, regenerative potential, and antimicrobial activity [162,163,195,196]. Among these, chitosan is of special interest due to its established medical applications. However, studies specifically focusing on the incorporation of chitosan into endodontic sealers remain limited. Future research should aim to evaluate the physicochemical and biological behavior of chitosan within the root canal environment and develop optimized formulations that ensure effective integration with other sealer components. Of particular importance are investigations into chemical modifications of chitosan, the incorporation of nanoparticles, and its potential influence on sealing durability and periapical tissue healing. The incorporation of chitosan into bioactive silicate-based materials could facilitate the development of intelligent endodontic materials with tailored therapeutic functionalities.

This direction holds particular promise in pediatric dentistry, where research on this topic remains scarce. Root canal filling materials used in primary teeth differ substantially from those employed in permanent teeth and currently represent a relatively small category. Given these distinctions, the combination of resorbable pastes with biopolymers—especially chitosan, constitutes a highly promising area for further investigation. Chitosan’s antibacterial properties, biodegradability, and low toxicity make it especially attractive for endodontic treatment in primary teeth. However, the existing literature provides only limited data in this field, highlighting a significant research gap. Expanding studies on the application of chitosan in pediatric endodontics could greatly enhance our understanding of its interactions with current materials and offer valuable insights into potential clinical benefits and long-term safety.

The improvement of the physicochemical properties of endodontic sealers will depend on multiple factors, including the synthesis method, the type and concentration of additive substances, and the form in which they are incorporated into the final product. Future development should focus on optimizing these parameters and conducting comprehensive evaluations of tissue responses in long-term, clinically relevant models that account for the dynamic conditions of the oral environment. Importantly, any modifications to existing materials should not compromise their well-established functional properties. Only a holistic approach—balancing innovation with safety and efficacy—can lead to the development of advanced sealers that maximize the success of endodontic treatment and support long-term oral health.

## Figures and Tables

**Table 1:** Examples of root canal sealer products currently available on the dental market, together with the advantages and disadvantages of each group.

Type of Sealers	Advantages	Disadvantages	Product Name (Manufacturer, Country)	Composition	References
Zinc oxide-eugenol based	Antimicrobial, resorbable when overextended, removable	Cytotoxicity, porosity, shrinkage	Tubli-Seal (Kerr, Brea, CA, USA); Endomethazone N (Septodont, Saint-Maurdes-Fossés, France); Endoseal (Prevest, Jammu, India); Essenseal (Produits Dentaires, Vevey, Switzerland); Pulpdent Root Canal Sealer (Pulpdent, Watertown, MA, USA)	Zinc oxide, eugenol, barium sulfate, thymol iodide, eugenol, etc.	[24,25,26,27,28]
Glass ionomer based	Chemical bonding to dentin, low shrinkage, low solubility, fracture resistance	Short-term antimicrobial, difficult retreatment	Ketac Endo (3M ESPE, Saint Paul, MN, USA)	Glass powder, polycarboxylic acids, tartaric acid	[13]
Silicone based	Good biocompatibility, dimensional stability	Lack of antibacterial activity, cost	GuttaFlow 2; GuttaFlow Bioseal (Coltene Whaledent, Altstätten, Switzerland)	Gutta-percha powder, silicone oil, polydimethylsiloxane	[29]
Methacrylate resin based	Adhesion, monoblock potential, fracture resistance	Cytotoxicity, residual monomers, gaps, cost	EndoRez (Ultradent, South Jordan, UT, USA); Super-Bond RC Sealer (SunMedical, Shiga, Japan)	UDMA, TEGDMA, PMMA, MMA, 4-META	[24,30,31]
Epoxy resin based	Good sealing, low shrinkage, dimensional stability	Moderate biocompatibility, no bioactivity	AH Plus (Dentsply Sirona, Charlotte, NC, USA); Adseal (Meta Biomed, Chungcheongbuk-do, Republic of Korea); AH-26 (Dentsply Maillefer, Ballaigues, Switzerland)	Epoxy resin, amine adducts, zirconium dioxide	[32,33,34]
Calcium hydroxide based	Alkaline pH, biocompatibility, hard tissue induction	Solubility, weaker sealing	Sealapex (Kerr, Brea, CA, USA); Apexit Plus (Ivoclar Vivadent, Schaan, Liechtenstein); Calapex (Prevest, Jammu, India)	Calcium hydroxide, calcium oxide, bismuth oxide, resin components	[35,36,37]
Bioceramic based	Bioactivity, no shrinkage, chemical bonding to dentin	Difficult to remove, neurotoxicity risk	EndoSequence BC Sealer (Brasseler, Savannah, GA, USA); TotalFill BC Sealer (FKG, La Chaux-de-Fonds, Switzerland); BioRoot RCS (Septodont, Saint-Maurdes-Fossés, France); AH Plus Bioceramic (Dentsply Sirona, Charlotte, NC, USA); CeraSeal (Meta Biomed, Chungcheongbuk-do, Republic of Korea)	Tricalcium silicate, dicalcium silicate, calcium phosphate, zirconium oxide, tricalcium aluminate DMSO	[38,39,40,41,42,43]

**Table 2:** Characteristics of studies on the combination of endodontic sealers with CS discussed in this article.

Study	Aim	Materials and Methods	Key Results	Conclusions
Ratih et al. (2023) [185]	Evaluate antibacterial and cytotoxic effects of epoxy resin-based sealer with different CS concentrations	AH26 modified with 0%, 10%, 20%, 30% CS; *E. faecalis* diffusion test; Vero cell cytotoxicity assay	10% CS: largest inhibition zone; 30% CS: lowest cytotoxicity	CS increases antibacterial efficacy and decreases cytotoxicity in a concentration-dependent manner
Maharti et al. (2023) [186]	Compare tricalcium silicate–CS-based sealer with commercial sealers	TCS-C vs. AH Plus vs. Sure-Seal Root; physicochemical tests; fibroblast viability assay	TCS-C: good biocompatibility, moderate flow, comparable thickness	TCS-C has potential, though further optimization is needed
Pattanaik et al. (2020) [187]	Evaluate antifungal activity of sealers with and without 2% CS	AH Plus, Apexit Plus, MTA Fillapex with and without 2% CS; *C. albicans* disk diffusion	Sealers and CS showed higher antifungal activity; AH Plus and CS was most effective	CS improves antifungal efficacy of all sealer types
Rane et al. (2023) [188]	Assess apical microleakage of bioceramic sealer with/without CS	Bioceramic sealer with and without CS; dye leakage test on extracted teeth	Lower microleakage in CS-modified group	CS improves sealing performance and dentinal adhesion
Harishma et al. (2024) [189]	Measure push-out bond strength of sealers with and without CS	Adseal and CeraSeal with and without 2% CS; tested at 7 mm and 11 mm from apex	Higher bond strength in CS-modified groups at both levels	CS enhances adhesion of sealers to dentin
Loyola-Rodríguez et al. (2019) [190]	Compare antibacterial activity of sealers modified with CS, AgNPs, and CHX	Multiple sealers with CS-CHX, AgNPs, Ca(OH)_2_, and CHX; direct *E. faecalis* inhibition test	Highest activity for CS-CHX modified groups	CS-CHX is an effective synergistic antibacterial modification

Abbreviations: All abbreviations used in this table are listed at the end of the article.

**Table 3:** Advantages and disadvantages of various types of endodontic sealers.

Type of Sealers	Advantages	Disadvantages
Zinc oxide-eugenol based	strong antimicrobial activity, sufficient working time, these sealers are absorbed if pushed into the periapical tissues, the sealer can be removed during root canal re-treatment with a combination of mechanical means and chloroform solvent,	cytotoxic considering the composition of eugenol, porous and subject to shrinkage, resulting in reduced material dimensions,
Glass ionomer based	chemically bond to the dentin structure, low shrinkage, low solubility in the presence of tissue fluids increased resistance to vertical root fracture,	short time antimicrobial activity of Ketac-Endo sealer, very difficult to remove it from the canal during endodontic retreatment,
Silicone based	short setting time, no cytotoxicity, dimensional stability, good biocompatibility,	lack of antibacterial activity, high cost of preparations,
Methacrylate resin based	good adhesion properties to root dentine, monoblock concept additionally improves sealing, increased resistance of root fracture,	cytotoxic, can cause gaps along the dentine/sealer interface, presence of residual monomers that may cause mutagenicity and genotoxicity, high cost of preparations,
Epoxy resin based	low polymerization shrinkage, good sealing, dimensional stability, low solubility, long working time, good adhesion to the canal walls,	presence of the sealer outside the apical opening may lead to chronic inflammation, moderately biocompatible—in contact with tissues they may cause a mild inflammatory reaction, these reactions usually decrease over time, lack of bioactive properties—epoxy sealants do not induce the formation of new mineral structures,
Calcium hydroxide based	good biocompatibility, osteo- and cementogenic potential, strong alkaline effect (pH approx. 12.5), which helps eliminate microorganisms and neutralize bacterial endotoxins,	high solubility, loss of material dimensions in the root canal over time, worse sealing properties compared to sealers based on epoxy resin and bioceramics,
Bioceramic based	high bioactivity, high biocompatibility, no shrinkage during setting, hydrophilic and tolerates moist environment well, compared to epoxy sealers, bioceramic sealers show lower cytotoxicity and lower susceptibility to dissolution, in comparison with calcium hydroxide-based sealers—bioceramics offer better volume stability and clinical durability, ability to chemically bond to dentin	difficult to remove during root canal retreatment, high cost of preparations, risk of neurotoxicity,

## Data Availability

No new data were created or analyzed in this study. Data sharing is not applicable to this article.

## Abbreviations

The following abbreviations are used in this manuscript:

4-META	4-Methacryloxyethyl Trimellitate Anhydride
α-TCP	α-Tricalcium Phosphate
AgNPs	Silver Nanoparticles
Bis-GMA	Bisphenol A Diglycidyl Ether Methacrylate
CHX	Chlorhexidine
CHX-HMP NPs	Chlorhexidine Nanoparticles Complexed with Hexametaphosphate
CS	Chitosan
CS-CHX	Chitosan–Chlorhexidine Complex
CS-NPs	Chitosan Nanoparticles
DMSO	Dimethyl Sulfoxide
EDTA	Ethylenediaminetetraacetic Acid
ERB	Epoxy Resin-Based Sealer
MMA	Methyl Methacrylate
MTA	Mineral Trioxide Aggregate
PMMA	Polymethyl Methacrylate
TCS-C	Tricalcium Silicate and Chitosan-Based Sealer
TEGDMA	Triethylene Glycol Dimethacrylate
UDMA	Urethane Dimethacrylate
ZOE	Zinc Oxide and Eugenol
